# Clinical features of anti-SAE1 antibody-positive myositis and interstitial lung disease: a multicenter, retrospective study in Taiwan

**DOI:** 10.3389/fimmu.2024.1482000

**Published:** 2024-11-07

**Authors:** Chao-Yang Hsiao, Shu-Chi Tseng, Chung-Yuan Hsu, Li-Chung Chiu, Li-Jen Su, Tien-Ming Chan

**Affiliations:** ^1^ Division of Rheumatology, Allergy and Immunology, Department of Internal Medicine, Chang Gung Memorial Hospital, and Chang Gung University, Taoyuan, Taiwan; ^2^ Department of Biomedical Sciences and Engineering, National Central University, Taoyuan, Taiwan; ^3^ Department of Medical Imaging and Intervention, Chang Gung Memorial Hospital at Linkou and Chang Gung University, Taoyuan, Taiwan; ^4^ Division of Rheumatology, Allergy and Immunology, Department of Internal Medicine, Chang Gung Memorial Hospital, Kaohsiung, Taiwan; ^5^ Department of Thoracic Medicine, Chang Gung Memorial Hospital, Chang Gung University College of Medicine, Taoyuan, Taiwan

**Keywords:** autoantibody, idiopathic inflammatory myopathies, interstitial lung disease, positive predictive value, autoimmune, anti-SAE1

## Abstract

**Introduction:**

The clinical characteristics of patients positive for anti-small ubiquitin-like modifier 1-activating enzyme subunit 1 (SAE1) antibodies and diagnosed with idiopathic inflammatory myopathies (IIMs) vary across different cohorts and ethnicities, particularly concerning interstitial lung disease (ILD). We aimed to assess the clinical utility of the line immunoblot assay (LIA) in detecting anti-SAE1 autoantibodies and evaluate the clinical relevance and chronology of ILD development in relation to SAE1 autoantibody positivity among Taiwanese patients.

**Methods:**

We retrospectively conducted a population-based cohort analysis involving 6,496 patients who visited Chang Gung Memorial Health System across Taiwan from May 2018 to December 2021. Patients were assayed for myositis-specific autoantibodies (MSAs) and myositis-associated autoantibodies (MAAs) using the LIA method, and the antinuclear antibody (ANA) indirect immunofluorescence (IIF) method was used to evaluate ANA patterns. Of these, 70 SAE1-positive patients (1.08%) were included and followed up until December 2023. Associations with clinical characteristics and final diagnosis, particularly ILD, were assessed.

**Results:**

Among the 70 SAE1-positive patients, 10 (14.3%) were strongly positive and 60 (85.7%) were weakly positive. In the strong positive group, 70% (7/10) were diagnosed with IIM, with most (5/7) showing a concordant ANA IIF pattern (speckled type). Six patients presented ILD either before (1/6) or after (5/6) IIM diagnosis; the majority (4/6) were classified as organizing pneumonia. The remaining 30.0% (3/10) had connective tissue disease (CTD) other than IIM without detectable ILD during follow-up, and none demonstrated a concordant ANA IIF pattern. In the weakly positive group, only 5.0% (3/60) had IIM and 3.3% (2/60) had ILD. The positive predictive value for strong positive SAE1 autoantibodies in diagnosing IIM was significantly higher than for weak positives (70.0% vs. 5.0%; *p* < 0.001).

**Conclusions:**

The study suggests that strong positive SAE1 autoantibodies detected via LIA are more closely associated with IIM compared to weak positive results. A high prevalence of ILD was observed among strong positive Taiwanese patients, indicating the need for prompt screening. Patients with weak positive or discordant ANA IIF results may represent false positives with a lower ILD risk.

## 1 Introduction

Idiopathic inflammatory myopathies (IIMs) are heterogeneous autoimmune diseases with multiple organ involvement, typically presenting a characteristic skin rash, muscle weakness, arthritis, and interstitial lung disease (ILD). Universally accepted IIM classification criteria remain undetermined and are evolving. IIM subgroups include dermatomyositis (DM), polymyositis (PM), clinically amyopathic dermatomyositis (CADM), overlap myositis (OM), immune-mediated necrotizing myopathy, sporadic inclusion body myositis, and antisynthetase syndrome (ASS) (1–4). Previous epidemiologic studies have reported incidence rates for IIMs ranging from 4.27 to 7.89 per 100,000 person-years and prevalence rates ranging from 9.54 to 32.74 per 100,000 individuals (5–7). However, epidemiologic studies on IIM remain challenging due to the historical use of various classification systems for diagnosis (8). Autoantibodies in patients with IIM, i.e., myositis autoantibodies (MAs), can be further classified into myositis-specific autoantibodies (MSAs), detected uniquely in two of three patients with IIM presenting specific phenotypes (9) among each MSA and mutually exclusive to one another. Moreover, myositis-associated autoantibodies (MAAs), encountered in IIM but not specific to IIM, can be found in other connective tissue diseases (CTDs) (10). Although some MSAs, such as anti-Mi-2, anti-TIF-1-gamma, and anti-NXP-2, have been reported to be linked to a lower risk of ILD, other MSAs, such as anti-melanoma differentiation-associated protein 5 (anti-MDA-5), anti-aminoacyl tRNA synthetase (ARS), or certain MAAs (e.g., PM-Scl, Ku, and Ro52), have been reported to be associated with an increased risk of ILD (11, 12). ILD can be the initial manifestation of IIM, with evidence showing that ILD precedes the signs and symptoms of myopathy in 7.2% to 37.5% of cases (11). Thus, international respiratory guidelines suggest including an extended myositis panel for MA detection during ILD screening and evaluation (12, 13). Furthermore, some myositis autoantibodies, such as Jo-1 and PL-12 among ARS and PM-Scl autoantibodies, have been reported to be associated with isolated ILD without myositis presentations (11, 13, 14). The autoantibody against the small ubiquitin-like modifier (SUMO) 1-activating enzyme subunit 1 (SAE1) is an MSA that has been previously found exclusively in patients with DM and identified as a DM marker (15). Prevalence of this autoantibody differs between Caucasian (6%–8%) and Asian (1%–3%) cohorts of patients diagnosed with DM (15–18). Patients with anti-SAE1 autoantibody typically present more skin than muscle involvement. However, their association with dysphagia, cancer, and other extramuscular manifestations, especially ILD, varied in different cohorts, even with conflicting results. In contrast to patients positive for anti-SAE1 autoantibody in Caucasians, typically presenting DM with relatively uncommon ILD (9, 15, 17, 19), those in Asians may present CADM with a high prevalence of ILD (16, 20–22). Given the high prevalence of ILD among Asian IIM patients who are positive for the anti-SAE1 autoantibody, it remains to be determined whether ILD screening is necessary for patients positive for the anti-SAE1 autoantibody, with or without IIM, especially within the Asian population.

Although immunoprecipitation (IP) is regarded as the gold standard for testing MAs, it is time-consuming and unavailable in most laboratories (23). The line immunoblot assay (LIA), a newly developed method regarded as an alternative, could detect multiple MAs simultaneously. Its diagnostic accuracy was considered comparative to the gold standard, especially when using high cutoff values (24–26). The clinical accuracy of each MA detected using LIA can vary considerably, ranging from very good to poor (24). Few studies have reported the clinical utility of LIA in detecting anti-SAE1 autoantibody and its impact on different clinical subsets, including patients with IIM or other CTDs without myositis in Asian cohorts, considering the low prevalence of anti-SAE1 autoantibody among Asians.

We conducted this retrospective cohort study to (1) investigate the clinical characteristics, including the chronology of clinical symptoms of patients positive for anti-SAE1 autoantibody using a commercially available LIA, and (2) elucidate the clinical relevance of ILD with SAE1 autoantibody positivity in Taiwanese patients.

## 2 Materials and methods

### 2.1 Study sample

The Institutional Review Board of Chang Gung Memorial Hospital approved this study (protocol no. 202101542B0C103). Serum samples from patients who visited the Chang Gung Memorial Health System throughout Taiwan (composed of a network of seven hospital branches located in Linkou, Taipei, Taoyuan, Keelung, Yunlin, Chiayi, and Kaohsiung), the largest healthcare system in the country, accounting for about one-tenth of the nationwide health services, between May 2018 and December 2021, and underwent testing for MAs under clinical suspicion of IIM or another CTD, were analyzed.

**Figure 1:**
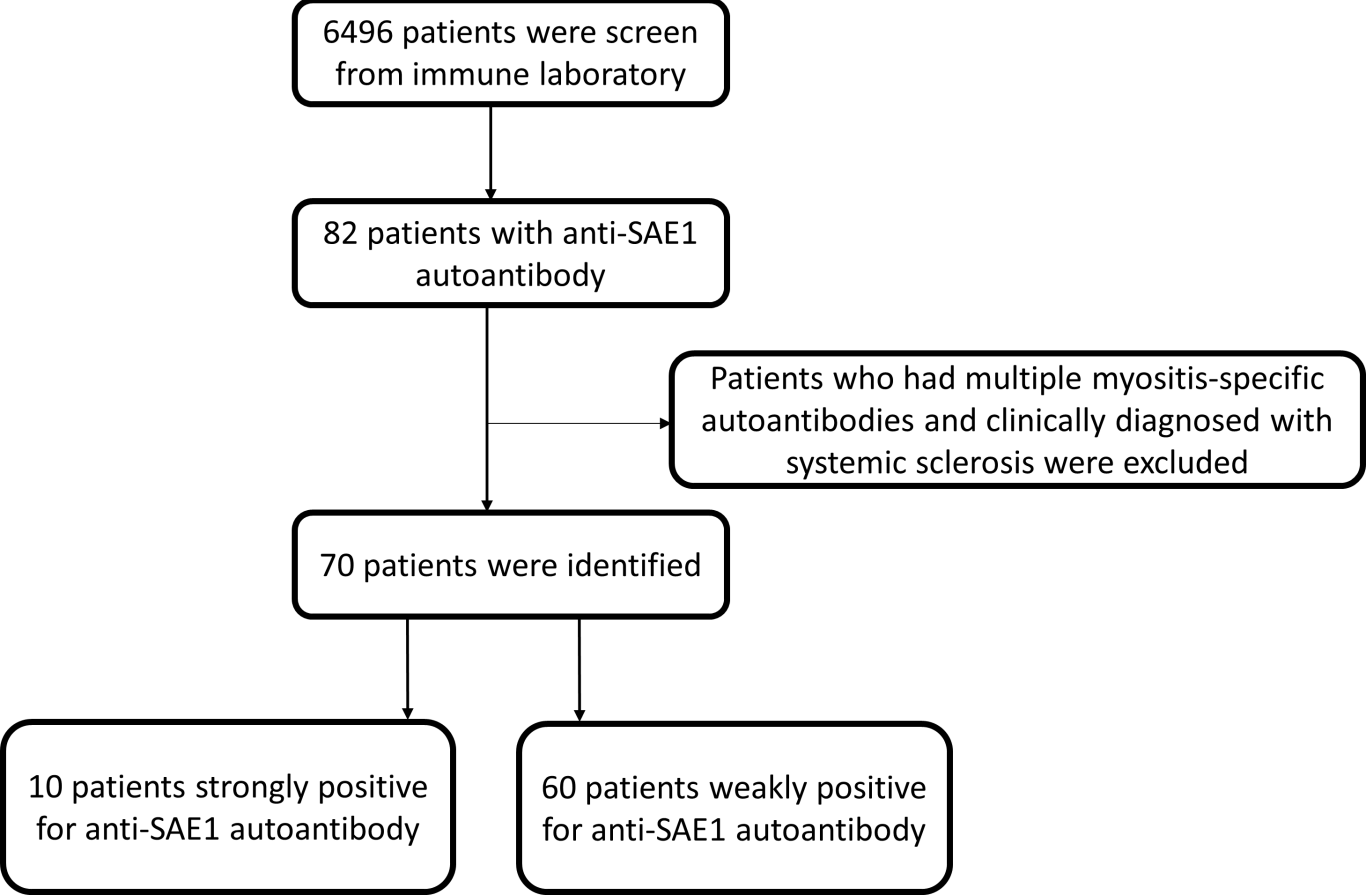
Selection flowchart.

A total of 70 patients (aged >18 years) with a detectable level of anti-SAE1 autoantibody screen from 6,496 myositis panel tests were included; they were further categorized into two subgroups: those strongly positive for anti-SAE1 autoantibody (*n* = 10) and those weakly positive for anti-SAE1 autoantibody (*n* = 60), as shown in **Figure 1**. The following patients were excluded: (1) those with multiple MSAs, and (2) those diagnosed with systemic sclerosis (SSc), except for cases of myositis concurrent with SSc. The first available data of MAs from each patient were analyzed with a clinical follow-up to December 2023.

### 2.2 Clinical data collection methodology

Retrospective chart reviews were conducted for the 70 patients with a detectable level of anti-SAE1 autoantibody to obtain clinical information and demographics, such as the extent of skin and muscle involvement, lung disease, cancer, and systemic clinical manifestations, when available. The diagnosis by the treating physician was recorded and categorized as “IIM” (including overlap syndromes), “CTD without myositis,” or “non-IIM/CTD.” IIM was diagnosed according to the Bohan and Peter criteria (1, 2), and CADM was diagnosed according to the Sontheimer criteria (27). Patients with other CTDs met the respective international criteria (4, 28–30). Diagnoses were verified by a review of extensive clinical information reflecting several years of care in each case.

### 2.3 LIA

LIA [EUROLINE Autoimmune Inflammatory Myopathies 16 Ag(IgG) test] was used to analyze the serum autoantibody profiles. LIA was conducted following the manufacturer’s instructions. The test kit provided a qualitative *in vitro* determination of immunoglobulin IgG human autoantibodies to 16 different antigens, namely, Mi-2α, Mi-2β, TIF1γ, MDA5, NXP2, SAE1, Ku, PM-Scl00, PM-Scl75, Jo-1, SRP, PL-7, PL-12, EJ, OJ, and Ro-52 in serum or plasma. Blot strips were digitalized using a camera, and band intensities were determined by a computer program (EUROLineScan, Euroimmun AG). Results were semiquantified and graded according to the manufacturer cutoff as negative = 0–5 units (U); borderline = 6–10 U; weakly positive (+) = 11–25 U; moderately positive (++) = 26–50 U; and strongly positive (+++) >50 U. The borderline results were considered negative according to the manufacturer’s recommendations.

In our study, we defined positive MAs as signal intensity >10 U (+, ++, or +++). Patients detected with a weakly positive signal intensity of anti-SAE1 autoantibody (11–25 U, +) via LIA were considered as a control group compared with those detected with a strongly positive signal intensity of anti-SAE1 autoantibody (>25 U, ++, or +++) because the weakly positive quantification was considered low specificity based on prior validation studies (25, 26).

### 2.4 Indirect immunofluorescence assay

The AESKUSLIDES kit (Aesku Diagnostics, Wendelsheim, Germany) was employed for the antinuclear antibody (ANA) assay, which was determined by indirect immunofluorescence (IIF) on the HEp-2 cell line. The test was performed according to the manufacturer’s instructions. We reviewed the IIF ANA patterns reported within 12 months of myositis autoantibody detection to improve diagnostic performance. An ANA titer of >1:80 was considered positive.

### 2.5 AtheNA Multi-Lyte ANA-II plus test system

Antiextractable nuclear antibodies (ENA: SSB/La, SSA/Ro, RNP, and Sm) were detected using the AtheNA Multi-Lyte^®^ antinuclear antibodies-II system. The test was conducted per the manufacturer’s protocol. We reviewed the antiextractable nuclear antibodies reported within 12 months of myositis autoantibody detection.

### 2.6 Definition of lung involvement and review of radiographic findings

ILD was defined according to clinical symptoms, pulmonary function testing, and the presence of inflammatory or fibrotic opacities on chest computed tomography (CT) or radiography. Disease onset was classified as ILD-preceding if the ILD diagnosis preceded the IIM diagnosis by >3 months, concomitant ILD if ILD and IIM were diagnosed in ≤3 months, or myositis-preceding if the IIM diagnosis preceded the ILD diagnosis by >3 months. The clinical course of ILD, according to the clinical presentation, was classified as acute (deteriorating <1 month from the onset of respiratory symptoms or the initial visit), subacute (deteriorating in 1–3 months), or chronic (stable or slowly progressive for >3 months). None of the patients had other known causes of ILD, and patients with pulmonary infection were excluded from the ILD category. A radiologist with expertise in ILD reviewed available chest CT images. The radiographic findings of interstitial patterns were classified as usual interstitial pneumonia, nonspecific interstitial pneumonia (NSIP), NSIP with organizing pneumonia (OP), OP pattern, and “unclassified” when there was an inability to classify ILD according to the current diagnostic framework according to the guidelines for idiopathic interstitial pneumonia (31, 32). A multidisciplinary discussion was conducted to resolve chest CT interpretation disagreements and reach a consensus.

### 2.7 Statistical analyses

The findings were presented in descriptive statistics, detailing frequencies and corresponding percentages. A parametric method (an independent *t*-test) was used to compare quantitative data, and the chi-square or Fisher’s exact test was used to compare proportions. We considered *p* < 0.05 as statistically significant. Data were analyzed with SPSS software (SPSS for Windows, version 22.0, Armonk, NY, USA).

## 3 Results

### 3.1 Demographic data of the cohort patients positive for anti-SAE1 autoantibody

Retrospective chart reviews of the 70 patients included in the study were conducted to obtain information on clinical characteristics. Most MAs were requested by rheumatologists (85.7%). Other requesting specialties included dermatologists (4.3%), respiratory physicians (4.3%), neurologists (2.9%), oncologists (1.4%), and an infectious disease specialist (1.4%). Among the 70 patients assessed, 10 had IIM, 43 had a CTD other than IIM, and 17 had no CTD.

Of the 70 patients assessed, 10 (14.3%) were strongly positive for anti-SAE1 autoantibody (>25 U), whereas 60 (85.7%) were weakly positive for anti-SAE1 autoantibodies (11–25 U) and were considered as the control group. Among the 10 individuals who tested strongly positive, 7 met the criteria for IIM and 6 of them had detectable ILD (6/7, 85.7%), and 3 were diagnosed with CTD other than myositis.

Among the seven patients who tested strongly positive for the anti-SAE1 autoantibody and met the criteria for IIM, three were diagnosed with DM, two with CADM, one with PM, and one with OM in the context of concurrent myositis and SSc.

The detailed characteristics of the 10 patients strongly positive for anti-SAE1 autoantibody are shown in **Table 1**.

**Table 1:** Characteristics of 10 patients strongly positive for anti-SAE1 autoantibody.

Variable	Strong positivity (>25 U) for anti-SAE1 autoantibody, *n* = 10 (%)
Sex (female)	6 (60.0%)
Mean age in years (SD)	57.70 (8.42)
Race/Ethnicity: Asian	10 (100.0%)
Ever smoker	3 (30.0%)
Myositis-associated autoantibodies	
Ro-52	2 (20.0%)
Antinuclear antibody (ANA)	7 (70.0%)
Compatible ANA pattern (speckled type)	5 (50.0%)
Interstitial lung disease (ILD)	6 (60.0%)
IIM	7 (70.0%)
Clinical amyopathic dermatomyositis	2
Dermatomyositis	3
Polymyositis	1
Overlap myositis*	1
CTD other than IIM	3 (30.0%)
Sjogren’s syndrome	0
Systemic lupus erythematosus	0
Mixed connective tissue disease	1
Undifferentiated CTD	1
Spondyloarthritis	1
Non-CTD/IIM	0
Management for IIM patients	
Corticosteroid	0
Corticosteroid + immunosuppressants^**^	4
Immunosuppressants^**^	3
No treatment	0

IIM, idiopathic inflammatory myopathy; IIF, indirect immunofluorescent; ARS, antiaminoacyl tRNA synthetase; including EJ, OJ, PL-7, PL-12, and Jo-1; CTD, connective tissue disease; RA, rheumatoid arthritis; IPF, idiopathic pulmonary fibrosis; CTD, connective tissue disease; NA, not available.

*The patient with a diagnosis of overlap myositis was found to have coexisting myositis and systemic sclerosis (SSc).

**In this study, medications classified as “immunosuppressants” included methotrexate (MTX), azathioprine (AZA), mycophenolate mofetil (MMF), and cyclophosphamide (CYC).

In contrast, only 3 of the 60 patients who were weakly positive were diagnosed with IIM, and 1 of those 3 had detectable ILD (1/3, 33.3%). Additionally, one patient in the weak positive group was diagnosed with idiopathic pulmonary fibrosis (IPF) without IIM (not shown in **Table 1**).

Of 70 patients, 10 were classified as IIM, yielding an overall PPV of 14.3%. The PPV for weak positive SAE1 autoantibodies (3/60, 5.0%) in IIM diagnosis was significantly lower than strong positive SAE1 autoantibodies (7/10, 70.0%) (*p* < 0.001), as shown in **Supplementary Table S1**. When using even higher cutoff values of signal intensity (+++, >50 U), the PPV for strong positive SAE1 autoantibodies in IIM diagnosis was 100% (4/4), despite the small number of patients.

### 3.2 Laboratory and radiographic characteristics of 10 patients with IIM in the cohort

In total, 10 of 70 patients in the cohort met the criteria for IIM. The antibody profile of myositis-associated antibody, ANA, and anti-ENA antibody of the 10 patients who had IIM with or without ILD in the cohort is summarized in **Table 2**; 7 of the 10 patients were strongly positive for anti-SAE1 autoantibody, whereas the remaining 3 patients were weakly positive for anti-SAE1 autoantibody. Six out of the seven patients with IIM who tested strongly positive for anti-SAE1 autoantibody had positive ANA results, with titers ranging from 1:80 to 1:320. Among these six patients, five had a speckled pattern (titers of 1:80 to 1:320), while one had a homogeneous pattern with a titer of 1:80. In the strong positive group, the majority of IIM patients (five of seven) exhibited a speckled ANA IIF pattern consistent with SAE1 autoantibodies, whereas none of the IIM patients in the weak positive group showed this concordance.

**Table 2:** Laboratory and radiographic characteristics of 10 patients with IIM in the cohort.

No.	Sex	SAE1	ANA	ENA	MAAs	ILD pattern	Diagnosis
1	M	+++	Speckled (1:320)	Negative	Ro-52	NSIP	CADM
2	M	+++	Speckled (1:320)	Negative	Negative	OP	DM
3	F	+++	Homogeneous (1:80)	RNP	Negative	Unclassified	PM
4	F	++	Speckled (1:160)	Negative	Ro-52	OP	CADM
5	F	++	Speckled (1:1280)	Negative	Negative	NSIP with OP	OM
6	F	++	Speckled (1:80)	Negative	Negative	OP	DM
7	F	++	Negative	Negative	Negative	No ILD pattern	DM
8	F	+	Cytoplasmic (1:1,280)	Negative	Negative	Fibrotic NSIP with OP	DM
9	M	+	Homogeneous (1:1,280)	Negative	Negative	No ILD pattern	DM
10	F	+	Negative	Negative	Negative	No ILD pattern	DM

IIM, idiopathic inflammatory myopathy; ILD, interstitial lung disease; MAAs, myositis-associated autoantibodies; CADM, clinically amyopathic dermatomyositis; DM, dermatomyositis; PM, polymyositis; OM, overlap myositis; ENA, extractable nuclear antigen; NSIP, nonspecific interstitial pneumonia; OP, organizing pneumonia. "+": weakly positive signal intensity ranged from 11 to 25 U for anti-SAE1 antibody via line immunoblot assay method. "++": strongly positive signal intensity ranged from 26 to 50 U for anti-SAE1 antibody via line immunoblot assay method."+++": strongly positive signal intensity above 50 U for anti-SAE1 antibody via line immunoblot assay method.

In the six IIM patients strongly positive for anti-SAE1 autoantibody also displaying ILD, there were three patients with a CT imaging radiographic pattern of OP, one with NSIP, one with both OP and NSIP, and one with unclassified ILD pattern. ENA positivity was found in only one patient who tested strongly positive for anti-SAE1 autoantibody. This patient showed ENA positivity specifically directed against RNP, without signs of overlap syndrome.

### 3.3 Clinical characteristics of patients strongly positive for anti-SAE1 autoantibody who were diagnosed with IIM and ILD

Six out of the seven patients with IIM who tested strongly positive for anti-SAE1 autoantibody displayed detectable ILD (**Table 3**). The median age of IIM onset was 59.5 years (not shown in Table). Four of these patients had DM. Among them, three patients had the classic cutaneous manifestations of DM (heliotrope sign and/or Gottron’s sign/rash), and two patients presented the shawl sign rash and/or the V sign rash. Except for these classic skin rashes, one patient presented a diffuse dark red skin rash. Two of them exhibited typical DM with the skin rash preceding myositis. Myositis developed within the subsequent 6 months. However, the other two patients exhibited CADM, and the time interval from initial skin rash to the latest visit in the two patients with CADM were 12 and 24 months, respectively, without myositis developing during the follow-up observation period.

**Table 3:** Clinical characteristics of six patients strongly positive for anti-SAE1 autoantibody who were diagnosed with IIM and ILD.

Characteristic	Case 1	Case 2	Case 3	Case 4	Case 5	Case 6	Total (*n* = 6)
SAE1 signal intensity	+++	+++	+++	++	++	++	
Sex	Male	Male	Female	Female	Female	Female	M:F, 1:2
Heliotrope sign	No	Yes	No	No	No	Yes	33%
Gottron papules	Yes	No	No	Yes	No	Yes	50%
Gottron sign	Yes	No	No	Yes	No	Yes	50%
Mechanic’s hands	No	No	No	No	No	No	0%
V sign	No	Yes	No	Yes	No	No	33%
Shawl sign	No	Yes	No	No	No	No	17%
Diffuse erythema	No	Yes	No	Yes	No	No	33%
Dysphagia	No	Yes	No	No	No	Yes	33%
Muscle weakness	No	Yes	Yes	No	Yes	Yes	50%
Highest recorded CK (IU/L)	Normal	401	Normal	187	1,682	Normal	33%
Arthritis	No	No	Yes	No	No	No	17%
Malignancy	No	Buccal	No	No	No	No	17%
Raynaud’s phenomenon	No	No	No	No	Yes	No	17%
Respiratory symptom	No	DOE	DOE	No	DOE	No	50%
Other MAAs	Ro-52	No	No	Ro-52	No	No	NA
Diagnosis	CADM	DM	PM	CADM	OM	DM	NA
ILD pattern	NSIP	OP	Unclassified	OP	OP + NSIP	OP	NA
Chest CT finding	Subpleural ground glass opacities in bilateral lower lobes	Peribronchovascular and a subpleural band of consolidations	Subpleural reticulation, honeycombing, and traction bronchiectasis	Central ground glass opacity surrounded by peripheral consolidation (reversed halo sign)	Consolidations superimposed on a background of subpleural ground glass opacities and reticulation	Arcade-like bands of consolidation, suggestive of perilobular pattern	NA
Distribution	Lower/Peripheral	Diffuse/Peripheral	Upper-mid/Peripheral	Lower/Peripheral	Lower/Peripheral	Lower/Peripheral	NA
Fibrotic	No	Yes	Yes	No	Yes	No	NA
ILD clinical course	Chronic	Chronic	Chronic	Chronic	Chronic	Chronic	NA
Medication	Corticosteroid Azathioprine	Methotrexate	Mycophenolic acid Azathioprine	Corticosteroid Methotrexate Azathioprine	Corticosteroid Methotrexate Cyclophosphamide	Corticosteroid Methotrexate	
Manifestations at onset	S	S	L	S	M	S/M	NA
Time interval (months)*	12	17	0	22	50	7	Median, 9.5
Follow-up time (months)**	12	88	50	24	58	16	Median, 37

*Time interval between disease onset and ILD detected.

**Follow-up time between disease onset to the latest visit.

ILD, interstitial lung disease; IIM, idiopathic inflammatory myopathy; CK, creatine kinase; DOE, dyspnea on exertion; MA, myositis autoantibody; CADM, clinically amyopathic dermatomyositis; DM, dermatomyositis; PM, polymyositis; OM, overlap myositis; NSIP, nonspecific interstitial pneumonia; OP, organizing pneumonia; UIP, usual interstitial pneumonia; S, presented skin disease first; L, presented lung disease first; M, presented muscle disease first; S/M, presented skin and muscle diseases; NA, not available. "++": strongly positive signal intensity ranged from 26 to 50 U for anti-SAE1 antibody via line immunoblot assay method. "+++": strongly positive signal intensity above 50 U for anti-SAE1 antibody via line immunoblot assay method.

Notably, one female patient aged between 70 and 75 years initially presented with ILD and was classified as having interstitial pneumonia with autoimmune feature (IPAF) with strong SAE1 autoantibody detection at disease onset but then developed myositis 1 year later and re-classified as PM. The patient exhibited a discordant ANA IIF pattern (homogeneous type) and was treated with immunosuppressants. The patient was initially treated with azathioprine but developed hepatitis, leading to a subsequent switch to mycophenolate mofetil.

The female patient aged between 50 and 55 years classified as having OM in the context of concurrent myositis and SSc presented with puffy fingers, digital tip ulcer, Raynaud’s phenomenon, fluctuating muscle weakness, and concordant ANA IIF pattern (speckled type) and was treated with intermittent high-dose corticosteroids and immunosuppressants for ILD and myositis control.

All six patients (100%) were diagnosed with ILD via available chest CT. Peripheral and basal lung involvement were seen in the majority of patients. Three of the six patients had respiratory symptoms. Two patients with available pulmonary function tests showed restrictive lung disease (data not shown). All six patients had ILD with a chronic or stable disease course. Overall, the median time interval between the disease onset and ILD detection by chest image was 9.5 months (0–50 months). Only one IIM patient strongly positive for anti-SAE1 autoantibody had malignancy (early-stage buccal cancer).

### 3.4 Clinical characteristics of patients strongly positive for anti-SAE1 autoantibody who were diagnosed with CTD other than myositis

In the group of patients who tested strongly positive for anti-SAE1 autoantibodies, three were diagnosed with CTD other than myositis: one with MCTD, one with UCTD, and one with spondyloarthritis.

The male patient aged between 60 and 65 years classified as having MCTD had an underlying disease of hepatitis C cirrhosis and presented with puffy fingers, pleuritis, lymphadenopathy, and mild sclerodactyly and tested positive for anti-U1-RNP antibodies, but with a low titer of ANA (homogeneous type). This patient met the diagnostic criteria for MCTD proposed by Tanaka et al. (33) and is receiving ongoing treatment with hydroxychloroquine (HCQ) for rheumatic disease control.

The male patient aged between 35 and 40 years with UCTD exhibited intermittent polyarthralgia and tested positive for ANA with discordant IIF pattern (homogeneous type), and is also being treated with HCQ.

The male patient aged between 55 and 60 years diagnosed with spondyloarthritis was identified as having psoriatic arthritis and tested negative for ANA, and is undergoing treatment with sulfasalazine (SSZ) and topical corticosteroid. None of these patients developed IIM or ILD during the follow-up periods.

## 4 Discussion

This is the first retrospective study to focus on the clinical features of anti-SAE1 autoantibodies detected by LIA in different disease subsets, including Taiwanese patients diagnosed with or without IIM, and the relevance of ILD with anti-SAE1 autoantibody positivity.

Our study showed that individuals with strong positive results for SAE1 autoantibodies detected via LIA are more likely to be diagnosed with IIM and to present with ILD compared to those with weak positive results. The overall PPV of SAE1 autoantibodies in diagnosing IIM using the LIA method was low (10/70, 14.3%) in our cohort. However, the PPV for strongly positive SAE1 autoantibodies in diagnosing IIM was significantly higher (7/10, 70.0%) than that for weakly positive SAE1 autoantibodies (3/60, 5.0%; *p* < 0.001).

Several studies have found that MSA and MAA are much more strongly associated with IIM when present at high antibody levels compared to weak antibody levels. However, there are discrepancies in the diagnostic performance among different MSAs (24–26, 34). When focusing on the diagnostic performance of SAE1 autoantibodies detected via the LIA method, most studies have reported relatively small sample sizes, predominantly with Caucasian populations. Ghirardello et al. reported that the specificity and PPV of SAE1 autoantibodies detected via LIA for IIM diagnosis reached 100% in a large Italian cohort comprising 267 IIM patients, 55 healthy subjects, and 203 diseased controls, but did not report the proportion of patients positive for SAE1 autoantibodies (24). Platteel et al., in a Dutch cohort, reported two cases strongly positive for SAE1 autoantibodies in the IIM group (*n* = 187) and two patients weakly positive for SAE1 autoantibodies in the non-IIM group (*n* = 632), concluding that the PPV of SAE1 autoantibodies detected via LIA for IIM diagnosis was only 50% (35).

Notably, our cohort was tested using an extended myositis panel via the LIA method under clinical suspicion of having myositis or other CTDs, not restricted to the clinical suspicion of myositis alone. This broader approach may lower the pretest probability and could explain why the diagnostic yield of LIA was lower than that reported in previous studies (24, 34, 35).

Among our cohort, all patients with strong positive results for SAE1 autoantibodies and detectable ILD were diagnosed with IIM either before or after their ILD diagnoses. None of our patients who had strong positive results of SAE1 autoantibodies were finally diagnosed with isolated ILD, which is consistent with the findings reported in most previous studies among Asian populations (16, 18, 36). However, within the strong positive group, one IIM patient did initially present with ILD of an unclassified pattern and then developed myositis without dermatological symptoms 1 year later. This case suggests that patients positive for anti-SAE1 autoantibodies can exhibit ILD upon initial presentation without obvious extrapulmonary symptoms. To the best of our knowledge, only one case report described a patient positive for anti-SAE1 autoantibody without dermatological manifestations but with obvious muscle weakness, myocarditis, and fatal rapid progressive ILD (20). Whether this represents an atypical presentation of SAE1-positive IIM or if there were unmeasurable confounding factors contributing to the clinical picture in this case warrants further investigation. Additionally, two patients strongly positive for anti-SAE1 autoantibody in our cohort presented a typical DM rash (heliotrope sign, Gottron’s sign, and/or papules) initially but did not develop myositis the following year and were classified as having CADM, which is consistent with but less frequently reported in previous studies among Asian populations (21, 37).

Studies have reported that concordance between the ANA immunofluorescence pattern and immunoblot assay may improve the clinical specificity of immunoblot autoantibody testing for myositis (38). Among the 10 patients who tested strongly positive for anti-SAE1 autoantibodies, 7 were diagnosed with IIM, 6 of whom had detectable ILD. The majority of these ILD patients (five out of six) exhibited a concordant ANA IIF pattern, specifically the speckled pattern, which is typically associated with anti-SAE1 autoantibodies (39), indicating true-positive results. In contrast, none of the remaining three patients who tested strongly positive for anti-SAE1 autoantibodies but were diagnosed with CTD without myositis displayed a concordant ANA IIF pattern. Therefore, these cases were considered false positives.

Although a previous study found an association between anti-Ro-52 autoantibodies and ILD, potentially leading to worse outcomes in IIM patients (40), the two patients in our cohort who tested strongly positive for SAE1 autoantibodies with coexisting anti-Ro-52 autoantibodies did not exhibit a rapid progressive ILD disease course.

The OP pattern was the most common type of ILD in IIM patients positive for anti-SAE1 autoantibodies in our cohort. Of the six IIM patients strongly positive for anti-SAE1 autoantibody with detectable ILD, four (67.7%) had a radiographic pattern on CT imaging of OP (one patient had OP superimposed with NSIP). The other two patients had NSIP and unclassified pattern, respectively. This finding is consistent with that presented in previous studies across several ethnicities (25, 32). Moreover, all six IIM patients strongly positive for anti-SAE1 autoantibodies had relatively mild ILD and responded well to treatment, similar to the findings of previous studies conducted in Asian groups (16, 18, 21, 22).

This study is subject to several limitations. First, its retrospective design limits the analysis to data already recorded in medical records, potentially missing information not typically documented. The ability to detect significant differences was hindered by the relatively small sample size and the low number of IIM diagnoses. This underscores the importance of conducting larger collaborative studies to assess these rare conditions effectively. The selective administration of pulmonary function tests and chest CT or high-resolution CT scans, based on individual clinical indications, may lead to an underrepresentation of patients with mild respiratory symptoms. Moreover, the predominant use of chest CTs to exclude occult malignancy, rather than specifically for ILD investigation, might skew the ILD findings.

Considering these findings suggest a significant prevalence of ILD among IIM patients who test strongly positive for anti-SAE1 autoantibodies via LIA. Our results substantially extend previous reports that included a limited number of Asian patients positive for SAE1 autoantibodies detected via LIA. However, interpreting SAE1 autoantibody positivity via LIA requires caution due to potential false positives, particularly in the absence of typical symptoms of IIM or pulmonary symptoms of ILD, inconsistent ANA results, and low titers of SAE1 autoantibodies. Patients who were strongly positive for anti-SAE1 autoantibodies and satisfied the IIM criteria may present with DM or CADM, with ILD potentially occurring either preceding or following IIM diagnoses. The most prevalent radiographic pattern for ILD among anti-SAE1 autoantibody-positive IIM patients appeared to be OP. Among patients who were strongly positive for anti-SAE1 autoantibodies and initially presented with ILD, the possibility of subsequent IIM development should be considered.

## 5 Conclusion

The study shows that strong positive SAE1 autoantibodies detected via LIA have a higher association with IIM compared to weak positive results. There is a high prevalence of ILD in IIM patients who are strongly positive for SAE1 autoantibodies detected by LIA, particularly in the Taiwanese population, suggesting the need for prompt ILD screening. However, patients who test positive for SAE1 autoantibodies via LIA but have low antibody titers, a discordant ANA IIF pattern, or lack symptoms indicative of IIM may represent false-positive cases and suggest a low risk of developing ILD.

## Data Availability

The original contributions presented in the study are included in the article/**Supplementary Material**. Further inquiries can be directed to the corresponding author.

## Glossary

ANA: Antinuclear antibody
ARS: Anti-aminoacyl tRNA synthetase
ASS: Antisynthetase syndrome
CADM: Clinical amyopathic dermatomyositis
CI: Confidence interval
CK: Creatine kinase
CT: Computed tomography
CTD: Connective tissue disease
DM: Dermatomyositis
DOE: Dyspnea on exertion
ENA: Extractable nuclear antigen
IIF: Indirect immunofluorescence assay
IIM: Idiopathic inflammatory myopathy
ILD: Interstitial lung disease
IPF: Idiopathic pulmonary fibrosis
L: Presented lung disease first
LIA: Line immunoblot assay
M: Presented muscle disease first
MA: Myositis autoantibody
MAA: Myositis-associated autoantibody
MDA5: Melanoma differential-associated gene 5
MSA: Myositis-specific autoantibody
na: Not available
NSIP: Nonspecific interstitial pneumonia
OM: Overlap myositis
OP: Organizing pneumonia
OR: Odds ratio
PM: Polymyositis
RA: Rheumatoid arthritis
S: Presented skin disease first
SAE1: Small ubiquitin-like modifier 1-activating enzyme subunit 1
SD: Standard deviation
S/M: Presented skin and muscle diseases
SSc: Systemic sclerosis
SUMO: Small ubiquitin-like modifier
UIP: Usual interstitial pneumonia
