# Supplementary material for: Clinical features of anti-SAE1 antibody-positive myositis and interstitial lung disease: a multicenter, retrospective study in Taiwan
Source: Front Immunol. 2024 Nov 7;15:1482000. doi: 10.3389/fimmu.2024.1482000 (PMC11579618; doi:10.3389/fimmu.2024.1482000)
Supplement: Supplementary file 1 [file Table1.docx]

**Table S1.** Positive predictive value of anti-SAE1 autoantibody testing via line immunoblot assay for IIM

| Positive predictive value | | | | | *P*-value | | | |
| --- | --- | --- | --- | --- | --- | --- | --- | --- |
| All positive patients | 10/70 (14.3%) | |  |  | | | N/A | |
| Patients with weak positive SAE1 autoantibodies | 3/60 (5.0%) | Patients with strong positive SAE1 autoantibodies | | | | 7/10 (70.0%) | | <0.001 |

NA: not available
